# Single-Cell Transcriptomics Sheds Light on Tumor Evolution: Perspectives from City of Hope’s Clinical Trial Teams

**DOI:** 10.3390/jcm13247507

**Published:** 2024-12-10

**Authors:** Patrick A. Cosgrove, Andrea H. Bild, Thanh H. Dellinger, Behnam Badie, Jana Portnow, Aritro Nath

**Affiliations:** 1 Department of Medical Oncology & Therapeutics Research, City of Hope National Medical Center, Duarte, CA 91010, USA; pcosgrove@coh.org (P.A.C.);; 2 Department of Surgery, City of Hope National Medical Center, Duarte, CA 91010, USA; 3 Division of Neurosurgery, City of Hope National Medical Center, Duarte, CA 91010, USA

**Keywords:** single-cell RNA sequencing, tumor heterogeneity, tumor evolution, precision medicine, clinical trials, breast cancer, ovarian cancer, glioblastoma

## Abstract

Tumor heterogeneity is a significant factor influencing cancer treatment effectiveness and can arise from genetic, epigenetic, and phenotypic variations among cancer cells. Understanding how tumor heterogeneity impacts tumor evolution and therapy response can lead to more effective treatments and improved patient outcomes. Traditional bulk genomic approaches fail to provide insights into cellular-level events, whereas single-cell RNA sequencing (scRNA-seq) offers transcriptomic analysis at the individual cell level, advancing our understanding of tumor growth, progression, and drug response. However, implementing single-cell approaches in clinical trials involves challenges, such as obtaining high-quality cells, technical variability, and the need for complex computational analysis. Effective implementation of single-cell genomics in clinical trials requires a collaborative “Team Medicine” approach, leveraging shared resources, expertise, and workflows. Here, we describe key technical considerations in implementing the collection of research biopsies and lessons learned from integrating scRNA-seq into City of Hope’s clinical trial design, highlighting collaborative efforts between computational and clinical teams across breast, brain, and ovarian cancer studies to understand the composition, phenotypic state, and underlying resistance mechanisms within the tumor microenvironment.

## 1. Introduction

Tumor heterogeneity remains a significant influencing factor that limits effective cancer treatment [1]. This heterogeneity arises from genetic, epigenetic, and phenotypic variations among cancer cells, leading to differential responses to therapy and contributing to disease relapse and metastasis. Understanding how tumor heterogeneity impacts tumor evolution, as well as response to therapies, can lead to more effective treatments and improved patient outcomes [2]. Traditionally, cancer evolution was thought to be driven by either the selection of a subpopulation of cancer cells with beneficial somatic alterations or the acquisition of new alterations that increase the fitness of cells in the face of selection pressure of treatment [3]. However, it is now increasingly being accepted that tumor evolution is likely fueled by significant contributions from non-genetic mechanisms [4], as well as the complex interplay of cell–cell communication between various components of the tumor microenvironment (TME) that contribute to tumor evolution, drug resistance and disease progression [5].

The tumor microenvironment (TME) consists of multiple cell types influenced by genetic and epigenetic changes that promote tumor growth, survival, and immune evasion. The TME can suppress immune responses while promoting tumor growth signals through complex interactions among its cellular components. Understanding the phenotypes and response mechanisms at the cellular level is crucial to uncovering the underlying mechanisms leading to resistance, immune activation, and response. Cell type diversity within the TME significantly influences patient responses to treatment. Tumors can be categorized as immune ‘hot’ or ‘cold’ based on the abundance of immune cells, such as macrophages and T-cells. The presence of fibroblasts, macrophages, monocytes, and T-cells contributes to the TME’s complexity. Additionally, resistance mechanisms can arise from autocrine feedback within cancer cells, affecting their sensitivity or resistance to treatment [6,7].

Traditional genomics approaches, such as bulk tumor RNA or DNA sequencing, are often implemented in clinical trials to provide a high-level overview of the tumor’s state. Bulk RNA-Sequencing and DNA-Sequencing have been vital to identify differentially expressed genes and aid in the development of potential biomarkers, as well as understand the genetic and epigenetic composition of the cancer development cancer diagnosis and leading to actionable treatment plans [8]. However, these methods are limited in their ability to offer insights into the cellular-level events within the tumor [9,10]. As such, bulk sequencing methods are also unable to provide detailed insight related to tumor heterogeneity [11,12]. Single-cell RNA sequencing (scRNA-seq) and single-nuclei RNA sequencing (snRNA-seq) provide transcriptomic analysis of RNA transcripts at the level of individual cells. This allows for insights into understanding tumor heterogeneity and subclonal diversity of cancer cells within tumors, identifying cell-type-specific responses to therapeutic treatment and tumor evolution, as well as characterizing phenotypic states contributing to immune suppression and cancer cell growth at a cellular level within the TME [13,14,15,16,17]. The use of single-cell genomics has significantly advanced our understanding of the mechanisms underlying tumor growth, progression, and drug response [10,18,19]. However, implementing single-cell approaches in clinical trials involves several challenges. For example, acquiring adequate high-quality viable cells from patient biopsies is often challenging. The process of dissociating tissues into single cells without altering their gene expression profiles demands meticulous handling and optimized protocols. Furthermore, scRNA-seq techniques can introduce technical variability, such as differences in cell capture efficiency, library preparation, and sequencing depth. This variability can impact the reproducibility and comparability of results across different studies and clinical sites, necessitating complex computational pipelines for accurate analysis [20,21].

Effective implementation of single-cell genomics in clinical trials and translational research therefore necessitates a collaborative approach, wherein translational research teams with expertise in computational biology and bioinformatics work closely together with medical oncologists and surgeons. By leveraging shared resources, expertise, and established workflows, this “Team Medicine” strategy aims to advance genomic understanding and improve patient outcomes. Here, we describe key technical considerations, perspectives, and lessons learned from integrating scRNA-seq and genomic studies into City of Hope’s clinical trial design. We also highlight collaborative efforts addressing questions across clinical network teams specializing in breast, brain, and ovarian cancer.

## 2. Building Robust Single-Cell Processing Pipelines for Implementation in Clinical and Translational Research

Through our collective efforts, we have performed snRNA-Seq and scRNA-Seq on over 645 samples from 250 patients in the past 7 years (2017–2024). These single-cell genomics studies have been published [22,23,24,25] or are in the process of being published. They include publicly available datasets encompassing patient cohorts from breast, brain, and ovarian cancers at City of Hope National Medical Center, as well as through external collaborations with the University of Kansas Medical Center and the Peter MacCallum Cancer Centre.

Implementing single-cell genomics in clinical trials requires careful consideration and integration of various factors into workflows to establish robust pipelines for research biopsy and malignant fluid collection and processing. This ensures sample quality, facilitates high-quality genomic studies, and minimizes the risk of sample degradation. The pipeline for single-cell genomic studies can be effectively divided into three key considerations: (1) tumor tissue availability, (2) tumor tissue collection and storage, and (3) single-cell processing.

### 2.1. Clinical Research Tumor Tissue Availability and Collection

Enrollment into clinical trials often requires the collection of clinical tumor biopsy samples as a condition of enrollment for standard-of-care clinical diagnosis and required testing. Beyond the scope of clinically required tissue biopsies, the limited availability of research biopsies poses a unique challenge to perform correlative genomic-focused studies. Although clinical research biopsies have the potential to improve the understanding of disease development and future treatment, the collection of additional research biopsies must take into consideration the potential scientific benefit while minimizing the risk to patients.

In 2019, the American Society of Clinical Oncology (ASCO) established an ethical framework of considerations related to categorizing expected scientific contributions, patient risk, and optional vs. mandatory research biopsy requirements [26]. As a confounding factor for consideration, the use of mandatory research biopsies has been shown to delay enrollment and raises concerns for patient exclusion from novel or investigative treatment and trials due to sufficient biopsies [27]. As a result, establishing clear informed consent of potential direct or indirect benefits to the patient, or lack thereof, can help to manage and temper patient expectations of potential outcomes [28,29].

The primary approach that we have adopted for collection of clinical research biopsy samples has been used by multiple clinical trials, which commonly seeks to limit the timepoint collections to a maximum of three timepoints including mandatory biopsy collection at screening/enrollment, and based on scientific goals, optional research biopsy collections within 9–12 weeks of trial initiation, and at end of trial or progression. Tissue collection is dependent on multiple factors including tumor size, treatment history, and cancer type with breast and ovarian cancers feasibly biopsied via core needle biopsy, while tissue collection for brain cancer collection is performed at surgical resection. In the event of surgical resection for breast, brain, and ovarian cancers, we have consistently used tissue requirements of 0.5–1 cm^3^ of tissue for single-cell genomic studies. The amount of tissue collection through imaging guided biopsy collection can range in size from 20-guage to 14-gauge core needle biopsies. However, the use of 14-gauge core biopsy has been selected for on-going and prospective genomic studies demonstrated through its accuracy and recovery of detectable tumor content and accuracy of ultrasound-guided tissue collection [30,31,32]. Additionally, we have found that 14-gauge core needle biopsies provide sufficient tissue to isolate sufficient nuclei from frozen tissue to facilitate snRNA-Seq and bulk DNA/RNA sequencing from the same tumor biopsy. Beyond solid tumor tissue collections, there is the opportunity for additional correlative studies to collect other sources of single cells from malignant fluids, such as pleural fluid, and ascites. Collection and banking of these tissues can be used for scRNA-Seq to understand disease progression; additionally, the sample can be collected ad hoc during the trial as available by the patient. Additionally, beyond an aliquot obtained for pathology and clinical diagnosis and treatment, these samples are largely discarded, but can provide a valuable source of viable cancer cells that can also be used to perform potential correlative studies in an in vitro setting assessing drug resistance [33].

### 2.2. Sample Preservation and Storage

**Figure 1:**
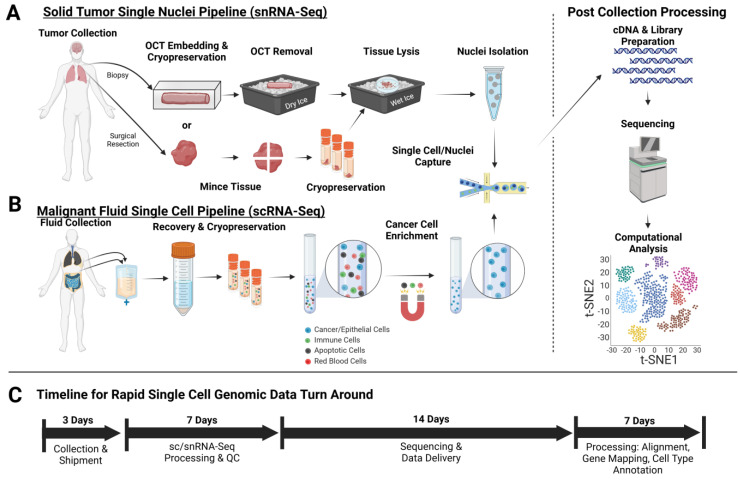
Single-cell processing pipelines. Overview of two pipelines established for the processing of clinical tumor tissue samples. (**A**) Solid tumor tissue taken from a core biopsy or surgical resection are placed in and cryopreserved in OCT embedding solution and stored frozen. Prior to snRNA-Seq, OCT is removed and tissue lysed before isolation of nuclei or (**B**) malignant fluids such as pleural fluid and ascites are collected from cancer patients and cryopreserved prior to scRNA-Seq. At the scRNA-Seq, cancer cells undergo magnetic cell depletion to remove immune cells, red blood cells, and dead cells. Lastly, (**C**) the sample-processing timeline is demonstrated to highlight the rapid data delivery of sc/snRNA-Seq data from clinical tumor collection within one month of the last timepoint collection. Created in https://BioRender.com/d14v636 (accessed on 9 December 2024).

Collection, preservation, and storage of solid tumor tissue samples are perhaps the most important considerations to preserve quality for scRNA-Seq, as the time to preservation is often cited as a potential source of technical variation and loss of sample quality, with desired maintenance of tissue samples at 4 °C and time to preservation under one hour from collection, while greater times pose a risk for RNA degradation and changes in gene expression profiles [34,35]. Groups including the Biorepositories and Biospecimen Research Branch have also highlighted procedures and best practices related to collection, processing, and storage of biospecimens to facilitate sample consistency and data quality [36]. Additionally, studies have shown that time from collection to storage and processing have significant negative impacts on cell viability and recovery of gene expression transcript detection and recovery [34,37]. Although many scRNA-Seq platforms use fresh dissociated tumor tissue as input material, that is often not feasible for clinical trial tissue collections due to technical capabilities, logistical coordination, longitudinal patient tumor collections, or multi-site and multi-patient collections [38,39]. As a result, there is a need to cryopreserve the tumor tissue for an extended duration prior to sequencing. To leverage this limitation, we have implemented two potential workstreams depending on the technical capabilities and resources of clinical collection sites to facilitate correlative studies utilizing scRNA-seq (Figure 1).

The primary pipeline and preservation method (Figure 1A) utilizes solid tumor tissues and embedding of the tumor tissue in cryomolds with Optimal Cutting Temperature (OCT) compound (Sakura Finetek, Torrance, CA, USA) prior to freezing on isopentane or dry ice. Embedding tumor tissue in OCT provides distinct advanta ges over traditional storage of tumor tissue as snap frozen or storage in cryopreservative buffers. First, the OCT acts as a protective barrier to the tumor tissue, allowing it to be stored long term in liquid nitrogen vapor phase while minimizing surface ‘freezer burn’ [40,41]. OCT-embedded tissue is also resistant to freeze–thaw effects during handling and sample processing to prevent tissue from thawing prematurely, such that when excess available tumor tissue is available, a portion of the tumor can be excised from the OCT while keeping the remaining tumor tissue frozen. Second, considering often restricted tissue availability, this unique approach to store tissue in OCT for snRNA-Seq applications allows for the preservation of any additional unused frozen tumor tissue while preventing the degradation of RNA/DNA from multiple freeze–thaw cycles by maintaining the tissue in a frozen state and enabling subsequent correlative studies such as bulk RNA-Seq, or WGS/WES [42]. Lastly, any remaining OCT-embedded tumor tissue can also be utilized directly for retrospective immunohistochemistry or immunofluorescent staining to characterize the TME composition or utilized for spatial transcriptomics to confirm the expression of potential biomarker candidates identified by scRNA-Seq [43,44], providing a unique opportunity to obtain paired single-cell and spatial imaging data to aid in understanding pathological analysis of the tumor tissue used.

As an alternative, in cases when freezing in OCT is not feasible, alternative methods include cryopreservation stored as 5 mm^3^ chunks of tissue stored in commercially available cryopreservative media such as CryoStor CS-10 from Stem Cell Technologies (Vancouver, BC, Canada). These commercially available reagents provide ready-to-use formulated preservatives allowing for minimal reagent preparation, longer shelf stability, and lack the need to prepare freshly prepared freezing media containing dimethyl sulfoxide, and Dulbecco’s Modified Eagle Medium (DMEM) or Roswell Park Memorial Institute (RPMI) 1640 Medium supplemented with fetal bovine serum (FBS) [45,46]. Consequently, these reagents can be utilized to minimize technical resources required at the site of collection. In addition, these reagents can be used to store viable single-cell suspensions isolated from malignant fluids such as pleural fluid and ascites (Figure 1B). Biobanking of malignant fluid samples, if present, is often necessary for medical purposes and largely discarded, but can be collected for potential correlative studies and processed alongside isolated nuclei from solid tumor samples from snRNA-Seq of to further study tumor evolution and drug resistance [47,48].

### 2.3. Single-Cell Processing

To facilitate downstream sample processing, the methods utilized for single-cell transcriptomics are dependent on the origin and type of the tumor tissue. Traditional scRNA-Seq is feasible from either dissociated fresh tissue or collected from malignant fluids such as ascites or plural fluid, while snRNA-seq is utilized for frozen and banked tumor tissue samples. snRNA-Seq currently accounts for 60% (388 samples) of the patient samples processed to date, while the remaining 40% (257) are from scRNA-Seq samples processed from malignant fluids such as abdominal fluids ascites, pleural fluid, and cerebrospinal fluid (CSF). Processing for scRNA-Seq is often straight forward; after thawing and washing cryopreserved single cells, single-cell suspensions can undergo quick column-free cell-type depletion to remove immune cell and dead cell depletion to enrich for viable cancer cell recovery prior to scRNA-Seq [49,50] (Figure 1B). The establishment of robust processing pipelines and training of laboratory staff is crucial to ensuring high-quality single-cell data by minimizing technical variation, minimizing batch effects, preventing error and sample loss, and decreasing processing time, all of which can complicate downstream analysis [51,52,53]. Technical factors such as proficient training on cell and nuclei isolation protocols can help to prevent human error in fast-paced and highly detailed multi-step protocols while advanced preparation and use of approved reagents and labware prior to processing can decrease processing time, increase accuracy, and improve cell recovery, leading to more uniform targeted depth. Historically, the timeline for generation of sequencing data has been slow and would take upwards of two to three months; however, through the optimization of processing pipelines, utilization of high-throughput sequencing instruments, and development of established computational analysis pipelines, we have been able to effectively reduce the data turn around to one month (Figure 1C).

For solid tumor tissue biopsies and resection tissue embedded in OCT, the isolation of nuclei for snRNA-Seq is a multi-step process whereby solid tumor can be excised from the OCT while being kept frozen, any residual OCT can be quickly washed off in an ice-cold PBS wash, and frozen tumor tissue can be directly lysed through Dounce homogenization or minced in nonionic detergent based lysis buffers (Figure 1A). Keeping the tissue frozen until immediately before lysis minimizes the potential for induction of transcriptional changes within the cells before transcripts are captured. It is important to highlight that during the lysis and nuclei washing, a variety of underlying technical factors can influence the efficiency of nuclei release, nuclei recovery, and yield. Concentrations of salts, non-ionic detergents, and components in the lysis and wash buffers of nuclei isolation are critical for snRNA-Seq and often include hypotonic buffers, also often used to aid cell lysis by causing swelling and rupture of the cytoplasmic membrane and nuclei release [50,54,55]. Other factors such as inclusion of nuclease free bovine serum albumin (BSA) to minimize nuclei aggregation, RNase inhibitors to inhibit RNase activity and degradation, and use of low binding plasticware, such Eppendorf LoBind Eppendorf tubes (Enfield, CT, USA), are critical to prevent nuclei loss and maximize nuclei recovery [53,56,57,58].

Multiple vendors have launched nuclei isolation kits but often fail to disclose details of the proprietary components, making it difficult to identify potential factors that may influence isolation and cell-type-specific recovery. Testing and validation of nuclei isolation methods remains a significant factor influencing the efficiency of tissue-specific variations on nuclei isolation protocols established throughout the field [50,59]. Conversely, technical considerations of sample preparation need to be taken into account when using intact single cells from solid tumors, such that attempts to dissociate and isolate intact single cells by enzymatic digestion can introduce additional variance into the sample quality and affect cell viability and introduce stress response, affecting gene expression and transcriptional signatures [60,61,62]. As such, scRNA-Seq is often limited to freshly isolated tumor tissues, which is often not feasible in a clinical-trial-based setting and therefore necessitates the use of snRNA-Seq to rapidly process and freeze the tumor tissue to maintain the highest quality tissue feasible.

## 3. Leveraging and Establishing Computational Resources

Successful integration of single-cell sequencing technologies into clinical research requires extensive collaboration with computational biologists, statisticians, and bioinformaticians. Our efforts benefited significantly from the essential contributions of our bioinformatics and computational teams. They played a pivotal role in managing and storing large volumes of raw data, utilizing City of Hope’s POSEIDON platform [63] to ensure secure and efficient data storage and handling. Additionally, our team of mathematicians and bioinformaticians developed specialized pipelines for pre-processing the raw data, creating novel analytical models to advance the interpretation of scRNA-seq data (https://github.com/U54Bioinformatics, accessed on 22 October 2024).

One of the major outcomes of this collaboration was the development of innovative predictive models for drug response, which represent a critical step towards clinical application (https://github.com/aritronath/ENDORSE (accessed on 6 December 2024) [64], https://github.com/aritronath/Everolimus_MLB (accessed on 6 December 2024) [65]). These models not only enhanced our ability to derive meaningful insights from processed scRNA-seq data but also laid the groundwork for future translational research efforts aimed at improving personalized therapeutic strategies. The cross-disciplinary collaboration between our computational experts and clinical researchers continues to be a key factor in the success of this project, driving advancements in the field of precision medicine.

## 4. Insights into Mechanisms of Tumor Evolution Response in Breast, Ovarian and Brain Cancers

Embracing the concept of team medicine, we have leveraged our expertise regarding scRNA-Seq and snRNA-Seq to help understand cancer progression, tumor evolution and drug resistance across multiple cancer types, including breast, brain, and ovarian cancers, within the City of Hope network and through external collaborations, and continue to integrate single-cell transcriptomics studies into ongoing clinical trials (Table 1).

### 4.1. Breast Cancer: The FELINE Clinical Trial

**Figure 2:**
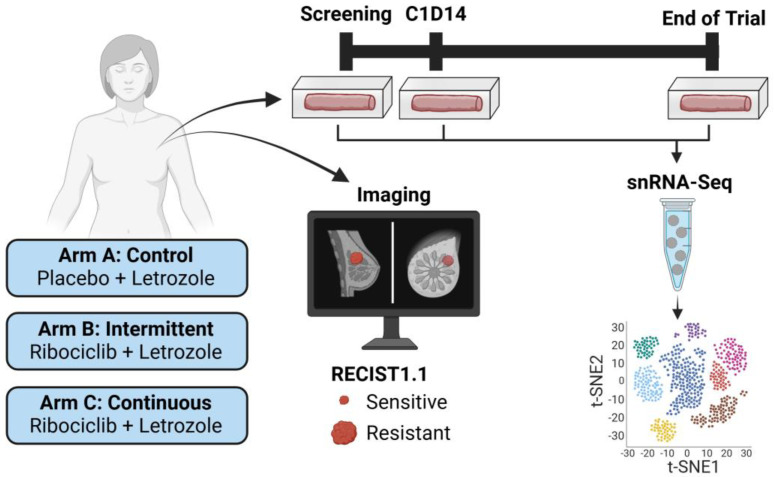
FELINE trial design. Early-stage ER+ breast cancer patients are stratified into one of three arms to receive letrozole alone, or combination therapy with ribociclib. Imaging of the tumor is obtained to classify patient response as sensitive or resistant based on RECIST criteria. Three core biopsies are obtained over the course of 6 months at screening, C1D14, and end of trial for snRNA-Seq. Created in https://BioRender.com/g23h600 (accessed on 9 December 2024).

For estrogen receptor (ER+) patients, the primary first-line treatment includes aromatase inhibitor endocrine therapies aimed at blocking estrogen synthesis and can be used as monotherapy or in combination with CDK4/6 inhibitors. To understand the mechanisms contributing to the evolution of resistance in early-stage breast cancer, we collaborated with Dr. Qamar Khan of the University of Kansas Medical Center (IND 127673) in a multi-center clinical trial (Femara (Letrozole) Plus Ribociclib (LEE011) or Placebo as Neo-adjuvant Endocrine Therapy for Women With ER-positive, HER2-negative Early Breast Cancer (FELINE), Clinical Trial # NCT02712723) [66,67] as a correlative study to perform single-nuclei RNA-sequencing on serially collected tumor biopsy samples from early-stage ER+ breast cancer patients treated with standard-of-care letrozole alone or in combination with ribociclib. A total of 120 patients were enrolled in the phase II clinical trial and 229 solid breast cancer core tumor biopsies samples were processed for scRNA-Seq from 77 patients, in which sufficient tumor samples were collected spanning three sequentially collected timepoints, including at screening (Day 0), after two cycles of treatment (C1D14), and at end of trial (Day 180, EOT) (Figure 2). The patient cohort for snRNA-Seq was analyzed as two sub-cohorts.

In the first publication, authored by Griffiths et al. 2021 [22], 95 samples from 41 patients were published as part of an exploratory cohort to study the evolution of resistant phenotypes in breast cancer cells. Through snRNA-Seq, the gene expression of identified phenotypic differences between sensitive and resistant tumors related to decreased estrogen receptor (ESR1) expression in resistant tumors was determined. With a loss of ER signaling in resistant tumors, JNK signaling as components of the MAPK network is elevated under combination treatment. Additionally, cancer cells that maintain estrogen signaling, whether under letrozole alone or combination ribociclib shift toward alternate proliferation methods through ERK upregulation through ERBB4 signaling.

In a follow-up study, authored by Griffiths et al. 2022 [23], single-nuclei sequencing data from the first sub-cohort were used in combination with snRNA-Seq data from an additional 30 patients and 90 samples in order to study treatment-related resistance mechanisms involved in communication between cancer cells and non-cancer cells within the TME. Reprogramming within the TME to promote cancer cell proliferation has been widely characterized through the suppression of immune responses, promotion of angiogenesis, and restructuring of the extracellular matrix to support cancer cell growth [68,69,70]. However, it has remained unknown how the tumor heterogeneity communication, tumor composition, and cell phenotypes and lead to the development of resistance under treatment conditions. In this study, three archetypes were identified based on the cell-type composition of the TME and categorized as (i) cancer-dominated, (ii) immune-enriched, and (iii) fibroblast-enriched. TME composition of sensitive and resistant tumors is distinct, with sensitive tumors being enriched with immune cell infiltration and not observed in resistant tumors. Next, communication networks were revealed between phenotypically different cell types as a measure of the ligand–receptor interactions amongst cell types within the TME, such that communication is mediated by ligand and matching receptor, altering the level of expression of transcriptomic profiles of sending and receiving cells through TWISTER (Tumor-Wide Integration of Signaling to Each Receiver). Cancer cells’ communication from resistant tumors promotes an immune suppressive phenotype leading the polarization of macrophages to an immunosuppressive phenotype and decreased communication between macrophages via IL-15/-18 (senders) and CD8+ T-cell receptors IL-2/15RA/18R, leading to decreased T-cell activation and cancer cell killing.

From a technical perspective, these studies also highlight one of the underlying features of performing scRNA-Seq, such that there remains inherent uncertainty of tumor purity obtained from biopsy samples and singl- nuclei quality recovered until after sequencing and post-analysis processing is complete. Significant factors were observed which contribute to recovery applications of high-quality nuclei, including biopsy tumor purity, number of cells recovered, proportion of mitochondrial gene expression as a measure of apoptotic or lysed cells, and total gene expression [71].

### 4.2. Phenotypic Plasticity in High-Grade Serous Ovarian Cancer (HGSOC)

HGSOC remains the most common type of ovarian cancer accounting for 70–80% of cancer deaths, and with an overall 49.1% 5-year survival rate, it remains the deadliest gynecological cancer [72,73]. Despite initial sensitivity to platinum-based chemotherapy, clonal diversity and evolution of resistance continue to provide a challenging landscape for the treatment of HGSOC [72,74]. Malignant fluids are often associated with advanced disease progression and poor prognosis, as the development of ascites is frequently associated with HGSOC. More than one third of patients develop ascites at the time of diagnosis, which allows for an opportunity for use in translational research to study the development of chemoresistance from temporally collected samples [75,76,77,78].

To investigate the transcriptional changes over the course of evolution of resistance at a cellular level, scRNA-Seq was utilized on isolated cancer cells obtained from malignant fluid collections of the peritoneal (ascites) and pleural cavities [24]. Collaborating with Dr. David Bowtell of the Peter MacCallum Cancer Centre, ascites samples were collected, and longitudinal malignant fluid of patients was collected for nine patients with annotated clinical treatment histories over the course of months to years. Unmatched samples were stratified into pre-treatment and post-treatment sample cohorts, and archetype analysis was used to identify transcriptional evolution of cells specializing in biological tasks. Three archetypes were determined, explaining transcription variation based on key gene expression identified at the vertices of each archetype and the diversity of evolution and specialization within cancer cells, with the identified archetypes including (i) metabolism and proliferation, (ii) DNA damage repair, and (iii) cell defense response. The metabolism and proliferation archetype was found to be enriched in glycolysis, and oxidative phosphorylation (OXPHOS), as well as proliferation pathways and associated key genes such as MK167 (proliferation), GAPDH (glycolysis) and enriched in unpaired post-treatment cohorts compared to treatment-naïve cohorts. The DNA damage repair archetype is enriched in apoptosis, P53, and TNFα, while the cell defense response phenotype is characterized by the enrichment of the interferon-ɣ response pathway and IL-6/JAK/STAT3 signaling pathways.

### 4.3. Brain Cancer Glioblastoma RAGE Inhibitors

Applications of scRNA-seq can be used to further understand mechanisms of immune responses and targeted treatments to overcome immune suppression. In glioblastoma patients, the use of anti-PD-1 immunotherapies as a neoadjuvant therapy can have reduced efficacy due to the administration of corticosteroids, often used to control cerebral edema and inflammation after post-surgical tumor resection [79,80,81].

To further understand the immune responses and regulation of immune response mechanisms, in collaboration with Dr. Behnam Badie of City of Hope, scRNA-Seq was performed on glioblastoma solid tumors obtained from five patients and single cells from resection cavity fluids taken 24 and 48 h post-surgical resection [25]. scRNA-Seq identified cell-type-specific expression of ligands S100A9, HMGB1, and S100B of receptor for advanced glycation end products (RAGE). RAGE is involved in the activation of inflammatory responses, including the expression of S100A9 by monocytes, while HMGB1 and S100B are involved in the activation of inflammation and are expressed in cancer and monocyte populations as an underlying mechanism contributing to inflammatory responses. Subsequent murine experiments demonstrated the clinical relevance of the inhibition of RAGE as a potential target for suppressing activation of inflammation.

## 5. Ongoing Team Efforts to Study Tumor Heterogeneity and Immunological Response in Ovarian Cancer and Glioblastoma

As we continue to utilize single-cell transcriptomics within the City of Hope network, ongoing clinical trials are in progress to investigate tumor heterogeneity, resistance, and mechanisms of immune response during cancer development (Table 2).

### 5.1. Pressurized Intraperitoneal Aerosolized Chemotherapy (PIPAC) in Ovarian Cancers

Treatment options for cancer patients with development of peritoneal carcinomatosis after first-line chemotherapy can be limited for those who cannot undergo surgical debulking. For cancers such as ovarian, uterine, gastric, appendiceal, and colorectal cancer, the use of pressurized intraperitoneal aerosolized chemotherapy (PIPAC) has recently arisen as a new treatment option for these patients. Aerosolized chemotherapy is administered into the abdominal cavity across three cycles every six weeks as part of an ongoing phase I clinical trial (NCT04329494, PI: Dellinger) [82,83] to evaluate the safety and efficacy of PIPAC treatment in ovarian, uterine, gastric, appendiceal, and colorectal cancer and snRNA-Seq is being performed as a correlative study to identify spatial and temporal mechanisms of cancer cell response to treatment, development of resistance, and tumor evolution by collecting solid tumor tissues prior to and post PIPAC treatment spatially across the peritoneal cavity over the course of PIPAC treatment. Solid tumor samples were obtained from each quadrant of the abdominal cavity via laparoscopic surgery at the time of PIPAC treatment at City of Hope under Institutional Review Board (IRB) # 19184.

Prospective collection and cryopreservative banking of PIPAC solid tumor tissue to date has resulted in collecting and cryopreserving 443 solid tumor and normal tissue samples from 27 unique patients across multiple cancer types for potential scRNA-Seq, with longitudinal collections of up to six PIPAC cycles and six to eight tumor samples per timepoint (0.5–1 cm^2^). The ongoing snRNA-Seq analysis of two PIPAC patients with low-grade serous ovarian cancer from this cohort is aimed at understanding the tumor heterogeneity and evolution of peritoneal metastases of patients undergoing PIPAC treatment to identify mechanisms of resistance and their impact on treatment outcomes.

### 5.2. Oncolytic Virotherapy in High-Grade Gliomas

Oncolytic virotherapy, which aims to selectively destroy tumor cells and elicit anti-tumor immune responses, faces challenges due to rapid immune-mediated clearance of oncolytic viruses (OVs). To address this problem, tumor tropic neural stem cells (NSCs) were genetically modified to carry a conditionally replicating adenovirus (CRAd-S-PK7; NSC-CRAd-S-pk7) [84]. The NSCs can protect the OVs from being destroyed by the host immune system while en route to tumor and enhance the OVs’ distribution within tumors. An ongoing multi-center phase 1 clinical trial of NSC-CRAd-S-pk7 (NCT05139056, PI: Portnow) is assessing the safety and feasibility of administering intracerebral doses of NSC-CRAd-S-pk7 in patients with recurrent high-grade gliomas. Participants undergo tumor resection, followed by injection of the agent into the surgical cavity and placement of a Rickham catheter in the surgical cavity to administer subsequent weekly doses of NSC-CRAd-S-pk7 as well as a second Rickham catheter in the lateral ventricle to obtain serial samples of CSF for correlative studies. Two weeks after the last intracerebral dose of NSC-CRAd-S-pk7 is administered, the study participants are taken back to the operating room to remove the Rickham catheters and obtain post-treatment tissue samples. In addition to determining the maximum tolerated number of weekly doses of NSC-CRAd-S-pk7, an important secondary objective is to assess its biologic activity.

Using scRNA-seq, our team is investigating the immunological responses in post-treatment tissue samples, as well as in tumor resection cavity and CSF. By collecting serial specimens from patients before and after treatment, we will identify the immunological states in the tumor microenvironment. We anticipate that patients who respond to treatment with NSC-CRAd-S-pk7 will show evidence of anti-tumor immune responses, including activation and infiltration of CD4+ and CD8+ T-cells, natural killer cells, reduced Tregs and a polarization of macrophages towards an anti-tumor phenotypic state.

## 6. Concluding Remarks

Implementation and use of single-cell RNA-Seq can bring a deeper understanding of the evolution and resistance of tumors at the cellular level. Applications of scRNA-Seq have made it feasible to identify heterogenous cell populations, characterize hallmark features of each cell type and study the impact of cell–cell communication within the TME [85,86,87]. Current applications of scRNA-Seq and snRNA-Seq are targeted at retrospective analysis of clinical research biopsies underlying mechanisms of resistance, susceptibility, and tumor evolution to develop new biomarkers and target key signaling pathways. Further, applications of single-cell genomics technologies aimed to advance precision medicine approaches are continuing to evolve at a rapid pace beyond single-cell transcriptomics (reviewed in [10]). For example, recent applications of single-cell DNA-sequencing (scDNA-Seq), single-cell-reduced representation bisulfite sequencing (scRRBS), and single-cell ATAC-seq (scATAC-seq), among others, aim to further advance efforts to understand both genetic and epigenetic contributions of the TME and cancer evolution [4,88].

As clinical trials continue to move toward a less invasive approach such as profiling patient tumor samples through the use of circulating tumor DNA (ctDNA) to identify actionable treatment therapies [89], there remains an opportunity to use and apply outcomes from scRNA-Seq proactively to identify potential resistance and susceptibility at a cellular level in real time to improve patient responses by leveraging biomarker development based on cell-type-specific responses. Although the direct use of single-cell transcriptomic applications for real-time patient-guided personalized medicine has yet to be realized, extensive validation will be required to translate their applications in clinical decision making. Nevertheless, there is potential for their utilization to guide the development of future precision medicine approaches [90,91,92]. The ability to integrate collection of tumor samples scRNA-Seq into our clinical trial framework has proven to be a valuable tool in supporting the discovery of the biology of multiple cancer types. By working with a team-based approach, we have gained novel understanding pertaining to the evolution and development of cancer resistance across multiple cancer types and indications.

## Figures and Tables

**Table 1:** Overview of completed transcriptomic studies investigating mechanisms of tumor evolution and response in breast, ovarian, and brain cancers.

Cancer Type	Study Design	sc/snRNA-Seq Key Outcomes	Works
Breast	snRNA-Seq of early-stage ER+ breast cancer patients treated with letrozole alone or combination ribociclib with longitudinal tumor biopsy collection at screening, C1D14, and end of trial (EOT)	- Decreased estrogen receptor signaling in resistant tumors	[22,23]
		- Increased JNK signaling under combination treatment	
		- Shift toward alternative proliferation through ERK upregulation and ERBB4 signaling	
		- Three archetypes of TME: cancer-dominated, immune-enriched, or fibroblast-enriched	
		- Polarization of macrophages toward immune suppressive phenotype and decreased communication with CD8+ T-cells	
Ovarian	scRNA-Seq of high-grade serous ovarian cancer patients with longitudinal malignant fluid collections	Identified three cancer cell archetypes:	[24]
		- Metabolism and proliferation archetype: enriched in glycolysis (GAPDH), OXPHOS and proliferation (MK167) pathways	
		- DNA damage repair archetype: enriched in apoptosis, p53, and TNFα	
		- Cell defense response archetype: Interferon-ɣ response and IL-6/JAK/STAT3 signaling pathways	
Brain	scRNA-Seq of resection cavity from glioblastoma patients collected at 24 h and 48 h post-surgical resection	- Identified cell-type-specific expression of S100A9, HMGV1 and S100B ligands of RAGE in cancer and monocyte populations involved in the activation of inflammatory responses	[25]

**Table 2:** Overview of active studies in brain and ovarian cancer patients.

Cancer Type	Study Design	Goals
Ovarian	snRNA-Seq of spatial and temporally collected peritoneal ovarian cancer tumors undergoing PIPAC treatment	- Understand tumor heterogeneity and evolution over the course of PIPAC treatment - Identify mechanisms of resistance related to treatment outcomes
Brain	scRNA-Seq and snRNA-Seq of serially collected tumor and fluid samples of glioblastoma patients treated with oncolytic virotherapy	- Identify immune responses in tumor samples, resection cavity and cerebrospinal fluids - Characterize phenotypes of T-cell and macrophage over the course of treatment

## Data Availability

No new data were created or analyzed in this study. Data sharing is not applicable to this article.
